# Voices of Vietnamese Workers in Japan: Content Analysis Using Free-Text Responses

**DOI:** 10.31662/jmaj.2025-0399

**Published:** 2025-11-21

**Authors:** Akihito Uezato, Nonoka Yoshizawa, Yui Fukuda, Pham Nguyen Quy, Soi Jeong, Takato Komatsu, Tadashi Yamashita, Ken Suzutani, Akane Hayakawa, Atsuko Taguchi

**Affiliations:** 1 Center for Basic Medical Research, International University of Health and Welfare, Tochigi, Japan; 2 Center for Medical Innovation, Institute of Science Tokyo, Tokyo, Japan; 3 Faculty of Nursing and Medical Care, Keio University, Tokyo, Japan; 4 Department of Medical Oncology, Kyoto Miniren Central Hospital, Kyoto, Japan; 5 Department of Psychiatry, Yokosuka Kyosai Hospital, Kanagawa, Japan; 6 Department of Psychiatry, Tokyo Metropolitan Bokutoh Hospital, Tokyo, Japan; 7 Department of Human Health Sciences, Graduate School of Medicine, Kyoto University, Kyoto, Japan; 8 Department of Psychiatry, Aizu Medical Center, Fukushima Medical University, Fukushima, Japan; 9 Tokyo Satsuki Hospital, Tokyo, Japan

**Keywords:** Japan, Vietnamese, migrant worker, mental health, content analysis

## Abstract

**Introduction:**

Japan’s aging population and declining birthrate have intensified the need for foreign workers, with Vietnamese workers now forming the largest group among over 2 million foreign laborers. While statistical studies on their work situations and mental health are increasing, they offer limited direct insight into these workers’ lived experiences. This study aimed to capture their firsthand voices, focusing on differences by residency status.

**Methods:**

Open-ended responses from 100 Vietnamese workers in Japan, collected through a 2022 survey, were analyzed. Content analysis was used to categorize experiences into positive and negative aspects.

**Results:**

Positive experiences included job satisfaction, adequate salaries, safe environments, supportive colleagues, and personal growth, with workers particularly valuing learning opportunities and workplace relationships that fostered development. Negative experiences, which were more prevalent, encompassed demanding workloads, unfair treatment, inadequate pay, long hours, and insufficient rest. Cultural and language barriers also hindered workplace relationships. Technical Intern Trainees (TIT) more frequently reported dissatisfaction with wages and conditions, while Engineer/Specialist in Humanities/International Services (ESI) workers more often cited interpersonal challenges linked to cultural adaptation.

**Conclusions:**

Vietnamese workers gain certain benefits from employment in Japan but frequently report dissatisfaction. TIT workers tend to face systemic issues, whereas ESI workers more often experience interpersonal challenges rooted in cultural differences. Addressing both types of challenges with consideration for the distinct situations of foreign workers may improve their mental health and well-being.

## Introduction

In Japan, a severe labor shortage has arisen due to the progression of a declining birthrate and aging population, as well as a decrease in the total population. To address this issue, the country has been promoting the acceptance of foreign workers. As of the end of October 2023, the number of foreign workers in Japan exceeded 2 million, representing nearly 3% of the domestic workforce, according to the Ministry of Health, Labour and Welfare ^(1)^. This is the highest figure recorded since mandatory reporting on the employment status of foreign workers began in 2007. By nationality, Vietnamese workers constitute the largest group at 25%, followed by those from China and the Philippines. The number of Vietnamese workers has increased significantly over the past decade.

Our previous studies have shown that a significant proportion of Vietnamese workers in Japan experience distress and are unable to seek help from healthcare professionals ^(2), (3)^. While numerous international and domestic studies have indicated that foreign workers are vulnerable to mental health issues, they provide limited direct insight into these workers’ lived experiences ^(4), (5), (6)^. Therefore, this study aimed to capture their firsthand voices, with particular attention to differences by residency status.

## Materials and Methods

### Participants

The study targeted Vietnamese individuals aged 18 and older who had work experience in Japan during the period from 2021 to 2022. Participants were recruited through posts on social media platforms conducted by the nonprofit organization TAIHEN Network (https://taihen.net/).

### Data collection

The present study was conducted as a secondary analysis of our previous survey ^(2)^, focusing on the open-ended responses to the question, “How do you feel about working in Japan?”. Data for the survey were collected via an online questionnaire conducted from August 6 to September 6, 2022. The questionnaire also included items on basic demographic information, psychological distress, and other related factors. Participants were informed about the study’s purpose and procedures and were required to provide informed consent before accessing the questionnaire. Only those who provided consent were able to proceed with the survey. The survey was conducted anonymously in Vietnamese.

### Content analysis

Out of 933 survey responses, individuals who provided no free-text responses, lacked information about their residency status, or did not appropriately answer the questions were excluded, leaving 100 respondents for the content analysis ^(7)^.

Vietnamese free-text responses were translated into Japanese by a bilingual researcher, and translation accuracy was verified through triangulation with other bilingual researchers. The responses were segmented into meaning units and inductively coded into positive and negative experiences. Two researchers independently coded and categorized the data; discrepancies were discussed and resolved by consensus, referring back to the original text when necessary. Provisional categories and subcategories were further reviewed by two additional researchers (four in total) until full agreement was reached, ensuring coherence and validity of the final categorization.

Finally, following previous studies, we compared the proportion of negative codes for each category of residency status―Student, Technical Intern Training (TIT), Specified Skilled Worker, and Engineer/Specialist in Humanities/International Services (ESI), the four main residency statuses of Vietnamese workers―using Fisher’s exact test.

### Ethics

The ethical review board of International University of Health and Welfare approved the study protocol (Numbers 19-Io-183).

## Results

### Demographics

The demographics of the target group are shown in Table 1. They consisted mostly of younger people in their 20s and 30s. The top four residency statuses were: TIT (39%), ESI (26%), Specified Skilled Worker (13%), and Student (6%). More than half of them worked in fields such as manufacturing, medical/nursing care, and construction (Table 1).

**Table 1.** Demographics (n = 100).

		n ( = %)
Age (years)	18-20	4
	21-25	31
	26-30	34
	31-35	20
	36-40	6
Sex	Male	48
	Female	49
	Other (including no response)	3
Status of residence	Permanent resident	3
	Long-term resident	0
	Spouse or child of Japanese national or permanent resident	2
	Technical intern training (TIT)	39
	Specified skilled worker	13
	Student	6
	Highly skilled professional	1
	Engineer/specialist in humanities /international services (ESI)	26
	Nursing care	5
	Designated activities	2
	Dependent	3
Field of work	Plant other than food plant	25
	Construction	10
	Medical/nursing care	16
	Food plant	7
	Computer engineer/IT	6
	Restaurants, food service	6
	Research/education	3
	Accommodation, hotel	1
	Sales, store, supermarket	2
	Cleaning	2
	Other	22

IT: information technology.

### Content analysis

From the free responses of 100 participants, the data was segmented into meaningful phrases, yielding 173 codes. Among these, 27 codes reflected positive experiences, while 146 codes reflected negative experiences. Below, the identified categories are numbered, with subcategories indicated by numbers in parentheses. Selected subcategories are illustrated with verbatim quotes (raw data) enclosed in quotation marks. Representative quotes for all subcategories are presented in Supplementary Tables 1 and 2.

#### I. Positive experiences

Twenty-seven codes were identified as positive experiences reported by Vietnamese workers in Japan. Analysis of these codes resulted in six categories and 15 subcategories (Table 2 and Supplementary Table 1). The categories included: 1. Satisfaction with work, 2. Adequate salary and rewards, 3. A conducive working environment, 4. Good interpersonal relationships and communication in the workplace, 5. Positive impacts on personal life, and 6. Miscellaneous.

**Table 2.** Categories and Subcategories for Positive Experiences.

**1. Satisfaction with work**
(1) Gained valuable learning experiences
(2) Work is comfortable relative to income
(3) Individual employees fulfill their responsibilities
(4) Work contributes to society
**2. Adequate salary and rewards**
(5) Receiving appropriate compensation
(6) Being able to send remittances to family
**3. A Conducive working environment**
(7) Work is supported by equipment
(8) The environment is safe and clean
**4. Good interpersonal relationships and communication in the workplace**
(9) Colleagues willingly offer support
(10) Not subjected to unfair treatment
(11) Colleagues show mutual care and consideration
(12) Having someone to consult with
(13) Overcoming cultural differences.
**5. Positive impacts on personal life**
(14) Enjoying recreational activities with colleagues to relieve stress
**6. Miscellaneous**
(15) The employer complies with labor laws

##### 1. Satisfaction with work

This category comprises the following subcategories: (1) Gained valuable learning experiences, (2) Work is comfortable relative to income, (3) Individual employees fulfill their responsibilities, and (4) Work contributes to society.

Within this category, subcategory (1) was the most frequently reported. Participants highlighted the growth and learning they achieved through their work in Japan:

“*I want to contribute to Japan because my time here has allowed me to grow. The Japanese people have taught me so much, and I’ve had many wonderful experiences in my life* (Specified Skilled Worker).”

These responses reflect the sense of personal and professional enrichment gained through a work-centered life in Japan.

##### 2. Adequate salary and rewards

This category consists of the following subcategories: (5) Receiving appropriate compensation and (6) Being able to send remittances to family.

##### 3. A Conducive working environment

This category consists of the following subcategories: (7) Work is supported by equipment, and (8) The environment is safe and clean.

##### 4. Good interpersonal relationships and communication in the workplace

This category consists of the following subcategories: (9) Colleagues willingly offer support, (10) Not subjected to unfair treatment, (11) Colleagues show mutual care and consideration, (12) Having someone to consult with, and (13) Overcoming cultural differences.

Within this category, subcategory (9) was the most frequently coded among positive experiences:

“*The salary is a bit low, but there are many kind people who are happy to help when I’m in trouble, and there’s no discrimination against foreigners* (Specified Skilled Worker).”

“*Even when the work is tough, I’m lucky to have colleagues who show mutual care and consideration. That’s why, despite the challenges, I want to work hard to be of help to others* (Nursing Care).”

“*My colleagues are very nice, friendly, and enthusiastic. They always help me when I’m in trouble. I want to improve my Japanese skills so I can communicate better with the people at work* (Student).”

These responses illustrate how supportive colleagues positively impact both work and life in Japan.

##### 5. Positive impacts on personal life

This category consists of the following subcategory: (14) Enjoying recreational activities with colleagues to relieve stress.

##### 6. Miscellaneous

This category consists of the following subcategory: (15) The employer complies with labor laws.

#### II. Negative experiences

A total of 146 codes were identified as negative experiences. An analysis of these codes resulted in six categories and 21 subcategories (Table 3 and Supplementary Table 2). The categories included: 1. Challenges and dissatisfaction with work, 2. Insufficient salary and rewards, 3. Inadequate rest, 4. Difficulties with workplace relationships and communication, 5. Negative impacts on personal life, and 6. Miscellaneous.

**Table 3.** Categories and Subcategories for Negative Experiences.

**1. Challenges and dissatisfaction with work**
(1) Being assigned work unfairly
(2) Work is overly demanding
(3) Frequent exposure to work pressure
(4) Job duties differ from expectations
(5) Mechanical and inflexible work methods
(6) Limited work assignment
(7) Unsafe working conditions
**2. Insufficient salary and rewards**
(8) Low wages
(9) Unpaid or insufficient wages
(10) Wage disparities in the workplace
(11) Inability to save due to Japan’s economic downturn
(12) Lack of access to welfare benefits
(13) Exclusion from shifts with better pay
(14) No salary increases
**3. Inadequate rest**
(15) Forced to work overtime
(16) Poor management of break times
(17) Unable to rest despite illness or fatigue
(18) Long working hours
(19) Insufficient personal time due to busy schedules
**4. Difficulties with workplace relationships and communication**
(20) Being treated unfairly due to being Vietnamese or a foreigner
(21) Lack of respect for one’s presence or opinions
(22) Challenges in communication and relationship-building due to language and cultural differences
(23) Mismatched dynamics with supervisors
(24) Experiencing violence
(25) Witnessing unfair treatment of others
**5. Negative impact on private life**
(26) Insufficient private time due to being busy
(27) Loss of personal freedom and privacy
**6. Miscellaneous**
(28) Lack of support or appropriate assistance in situations requiring help

##### 1. Challenges and dissatisfaction with work

This category comprises the following subcategories: (1) Being assigned work unfairly, (2) Work is overly demanding, (3) Frequent exposure to work pressure, (4) Job duties differ from expectations, (5) Mechanical and inflexible work methods, (6) Limited work assignment, and (7) Unsafe working conditions.

Concerning subcategory (1), workers reported being subjected to unfair workloads, both qualitatively and quantitatively:

“*Japanese people hate foreigners. … I was forced to do tough work for the first time. Vietnamese workers are made to do jobs that Japanese people don’t want to do. … Since coming to Japan, I’ve grown to dislike Japanese people* (TIT).”

“*I always have to do more work than Japanese people, and it’s very stressful* (TIT).”

For subcategory (2), participants described severe work conditions:

“*Despite paying a significant amount to come to Japan, I had to work at a company with low pay and hard labor* (TIT).”

“*The work is extremely difficult and exhausting. It makes me feel miserable and sad* (TIT).”

Psychological stress was a recurring theme in (3) Frequent exposure to work pressure:

“*I’m under so much pressure that I feel very tired. I’ve thought about returning home many times, but I’m bound by my contract and can’t leave. If I had the chance to choose a country to work in again, I would never choose Japan* (Student).”

“*Japan’s work culture is probably world-famous, but working with Japanese people makes me feel pressured, especially regarding meeting deadlines. I feel tense and pay extra attention to avoid making mistakes* (ESI).”

Many workers expressed frustration over discrepancies between promised and actual job roles in subcategory (4):

“*I was told I’d work in industrial packaging, but when I came to Japan, I was made to carry and transport wood. When I brought this up with the labor union, they said they couldn’t help me and called me a nuisance. When I spoke to the Japanese staff, I was sent back to Vietnam. I feel powerless in Japan* (TIT).”

“*The job I was told about during the interview was completely different from reality* (ESI).”

Workers reported unsafe environments and frequent accidents in (7) Unsafe working conditions:

“*The work is dangerous. Of the two Japanese people working with me, one tore a knee ligament, and the other fractured a rib* (TIT).”

“*We weren’t taught how to ensure safety at work when we joined. I’ve seen numerous work-related accidents happen at these companies* (ESI).”

##### 2. Insufficient salary and rewards

This category consists of the following subcategories: (8) Low wages, (9) Unpaid or insufficient wages, (10) Wage disparities in the workplace, (11) Inability to save due to Japan’s economic downturn, (12) Lack of access to welfare benefits, (13) Exclusion from shifts with better pay, (14) No salary increases.

Participants expressed dissatisfaction with inadequate compensation in subcategory (8):

“*I spent over 1 million yen to come to Japan, but the salary is low. I feel exploited and disappointed* (TIT).”

“*I hope foreign workers’ income will increase. Working in Japan feels like being just another machine* (TIT).”

In subcategory (9), instances of wage non-payment were highlighted:

“*I sometimes work overtime, but there is no overtime pay* (TIT).”

“*On rainy days, I don’t get paid* (TIT).”

Concerning subcategory (10), workers reported feeling unfairly compensated compared to their Japanese colleagues, despite contributing equally or more:

“*Even though I often work harder than Japanese colleagues, my pay is lower. Sometimes it makes me not want to work* (TIT).”

Workers’ financial struggles were expressed in subcategory (11):

“*Currently, the yen has dropped significantly, and my monthly expenses are high, leaving me with very little savings. Under such circumstances, I won’t choose to stay in Japan long-term* (ESI).”

##### 3. Inadequate rest

This category consists of the following subcategories: (15) Forced to work overtime, (16) Poor management of break times, (17) Unable to rest despite illness or fatigue, (18) Long working hours, (19) Insufficient personal time due to busy schedules.

Workers described feeling pressured to work beyond their scheduled hours in subcategory (15):

“*Foreign workers often face pressure both at work and in daily life. We’re discriminated against, and if we want to leave on time, we’re questioned or forced to work overtime* (Specified Skilled Worker).”

##### 4. Difficulties with workplace relationships and communication

This category comprises the following subcategories: (20) Being treated unfairly due to being Vietnamese or a foreigner, (21) Lack of respect for one’s presence or opinions, (22) Challenges in communication and relationship-building due to language and cultural differences, (23) Mismatched dynamics with supervisors, (24) Experiencing violence, and (25) Witnessing unfair treatment of others.

In subcategory (20), many participants described unacceptable treatment based on their nationality:

“*When there’s an issue between foreign workers and Japanese people, they always side with the Japanese. To the company I work for, we’re just cheap labor―nothing more, nothing less* (Specified Skilled Worker).”

“*If a Vietnamese worker makes a mistake, it’s announced during the morning meeting and mocked by the Japanese staff. But when a Japanese person makes a mistake, it’s ignored. We may be Vietnamese, but we are human too* (TIT).”

Regarding subcategory (21), participants recounted experiences where they felt disrespected without a clear reason:

“*No one listens to my opinions. Supervisors’ opinions are respected, but foreign workers receive no attention* (Nursing Care).”

“*I am suffering from bullying* (ESI).”

For subcategory (22), the difficulties stemming from cultural and language barriers were evident:

“*Because of cultural differences and age gaps, I often find it hard to speak up. Although I feel secure in my job, I have to be extremely careful about what I say or answer, so it’s not an environment where I can work freely and easily* (ESI).”

“*I try hard to get along with my colleagues, but Japanese people don’t express their thoughts. I have no idea what they think of me, so I’ve lost the desire to talk to them* (ESI).”

As for subcategory (24), although only one code was extracted, it depicted severe difficulties:

“*The manager often showed disdain toward us because we’re not Japanese. I experienced sexual harassment and was threatened with a knife* (Specified Skilled Worker).”

##### 5. Negative impact on private life

This category consists of the following subcategories: (26) Insufficient private time due to being busy, (27) Loss of personal freedom and privacy.

In subcategory (26), participants described lives dominated by work, leaving little room for personal fulfillment:

“*I wake up, go to work, and only close my eyes to sleep. Day after day, it’s just work―like being a robot. Life feels dull and unmotivating* (Specified Skilled Worker).”

“*I’m so busy that I don’t have time to have fun* (Student).”

Workers highlighted painful restrictions on their private lives in subcategory (27):

“*Please give us the right to live and protect our privacy. Don’t impose the Japanese way of living on us. We are human too. Give us freedom and equality. It shouldn’t matter where we go or who we live with as long as it doesn’t affect Japanese people’s work. Let us live freely and securely like you do* (TIT).”

##### 6. Miscellaneous

This category includes: (28) Lack of support or appropriate assistance in situations requiring help.

In subcategory (28), participants described feeling unsupported by those around them or even by institutions:

“*Japanese colleagues know that foreign workers are treated unfairly, but they don’t speak up* (ESI).”

“*When workplace injuries occur, we rarely receive proper compensation. Companies often hire lawyers to craft policies that shift the blame onto us, forcing us to admit fault so they can avoid responsibility. The Japanese judicial system often protects the interests of Japanese companies. I wish there was an organization that truly cared about the health, rights, and interests of foreign workers like us* (ESI).”

#### III. Comparison of the distribution of code counts for negative experiences by residency status

In the comparison of the distribution of code counts for negative experiences among the four main residency statuses, a significant difference was found between TIT and ESI (p = 0.031, Holm multiple comparison test): the categories “1. Challenges and Dissatisfaction with Work” and “2. Insufficient Salary and Rewards” showed a higher proportion of negative code counts for TIT compared to ESI, while in the categories “3. Inadequate Rest” and “4. Difficulties with Workplace Relationships and Communication”, ESI had a higher proportion of negative code counts than the TIT (Table 4). The distribution of code counts for positive experiences was not assessed due to the limited number of instances.

**Table 4.** Distribution of Code Counts for Negative Experiences.

Negative experience	Student	TIT	Specified skilled worker	ESI	Difference
1. Challenges and dissatisfaction with work	33.3%	34.2%	30.4%	22.6%	TIT > ESI
2. Insufficient salary and rewards	16.7%	28.8%	17.4%	9.7%	TIT > ESI
3. Inadequate rest	16.7%	0.0%	13.0%	12.9%	ESI > TIT
4. Difficulties with workplace relationships and communication	33.3%	31.5%	34.8%	45.2%	ESI > TIT
5. Negative impact on private life	0.0%	2.7%	0.0%	3.2%	n.s.
6. Miscellaneous	0.0%	2.7%	4.3%	6.5%	n.s.
Total counts of negative codes	6	73	23	31	

ESI: engineer/specialist in humanities/international services; n.s.: no significance; TIT: technical intern training.

## Discussion

Vietnamese workers gain certain benefits from employment in Japan but frequently report dissatisfaction. The characteristics of negative experiences appear to differ among major residency statuses, particularly between TIT and ESI statuses. In the following discussion, we examine the realities faced by Vietnamese workers in Japan, mostly focusing on these two residency statuses. By also considering their positive experiences, we explore ways to help them work more healthily and sustainably in Japan.

The TIT Program was launched in 1993 with the goal of contributing to international cooperation by transferring skills, techniques, or knowledge developed in Japan to developing regions, thereby supporting their economic development ^(8)^. However, the actual divergence between its stated purpose and its role as a labor force acceptance measure has been repeatedly pointed out, and the poor labor conditions, which can be considered human rights violations ^(9), (10)^. In fact, a report from 2022 by the Ministry of Health, Labour and Welfare indicated that 73.7% of businesses had engaged in illegal activities regarding TIT, highlighting that safety, appropriate wages, and working hours are often not adhered to ^(11)^. Furthermore, the current TIT Program restricts the freedom of labor mobility. Many TIT workers often come to Japan after taking on significant debt, and since they have no other means of repaying it other than working for the accepting company, they are prone to exploitation ^(12)^. Additionally, there are various restrictions impacting both work and private life, which affect mental health. These include being unable to bring family members over from their home country, being forced to live in shared dormitories, having unlawful restrictions on movement, and facing financial constraints ^(13)^. Amid these various limitations, it is not easy for trainees to voice their concerns.

In this study, TIT had the highest number of entries in the free-response section, suggesting that they are in circumstances where they have many realities they wish to convey. Many of the comments were accompanied by negative experiences, with numerous concerns about harsh working conditions in the workplace and inadequate wage payments. Additionally, some reported experiences where they felt they were not respected as human beings, and it was difficult to get help when they faced problems. These findings support previous research on TIT ^(14), (15), (16)^, but the continued reports of similar issues over several years indicate the need for ongoing discussions, not only within Japan but also in collaboration with sending countries, regarding the structure of the TIT Program. In 2024, the Japanese government decided to abolish the program and introduce a new system enabling long-term residency ^(17)^.

Individuals with the residency status of ESI are included in the category of so-called highly skilled foreign individuals, whose acceptance is being actively promoted. According to the Immigration Services Agency, highly skilled foreign individuals are defined as “high-quality talent that complements domestic capital and labor, and cannot be substituted,” as well as individuals “expected to bring innovation to Japan’s industries, promote the development of specialized and technical labor markets through mutual competition with Japanese professionals, and enhance the efficiency of Japan’s labor market.” Foreign workers under the ESI category include engineers in fields such as mechanical engineering, interpreters, designers, private-sector language teachers, and marketing professionals, all of whom are engaged in so-called white-collar jobs. The purpose of this status is to engage in activities that require technical knowledge or knowledge-based work or work that requires thinking or sensitivity based on foreign cultures, so simple labor is excluded from this category. Regarding wages, it is required that foreign workers in this category receive compensation equivalent to or greater than the amount Japanese workers would receive for the same job.

In this study, Vietnamese workers under the ESI category reported experiencing many issues with workplace relationships and communication. There were descriptions of struggles arising from strong pressure from Japanese colleagues and the difficulties of working with Japanese people who have a strict attitude toward accuracy and deadlines. Additionally, some comments suggested that communication difficulties arose from cultural differences. In this respect, Japanese conversations are often described as highly contextual, and the prevailing mode of thinking is considered more linear than dialectical ^(18)^. They often avoid emotional expression, as well as interpersonal conflict and confrontational language ^(19)^. Even compared to other culturally similar Asian countries, their approach to conflict resolution during disagreements is more subtle and indirect ^(20), (21), (22)^. These characteristics are among the most extreme globally, placing Japan in a uniquely distinct cultural position ^(23)^. Our previous study found that ESI workers reported the greatest distress among the major residency status categories. This finding was somewhat unexpected, as they generally occupy a relatively “privileged” social position and enjoy higher income levels. Their distress may, however, be attributable to the necessity of frequent interactions with Japanese colleagues and the demands of adapting to Japanese cultural norms.

In contrast, many workers across different residency statuses have reported positive experiences in the workplace. Previous studies on Japanese attitudes toward foreigners have suggested that while Japanese people tend to display favorable and tolerant attitudes toward Western countries, they are more likely to exhibit aversion and exclusionary attitudes toward Asian countries ^(24)^. However, since the 1990s, the proportion of Japanese people who feel a sense of affinity with Southeast Asians, including Vietnamese people, has been steadily increasing, reaching 71% in 2021, the same as their affinity toward Europeans ^(25), (26)^. This increase in the proportion of Japanese people feeling a sense of affinity is thought to be due to the growing contact between Southeast Asian workers living in Japan and the Japanese population. Among Asians, it can be said that Confucianism naturally facilitates the sharing of culture and values. For example, interpersonal relationships are often hierarchical and reciprocal, which are believed to maintain peace, harmony, and order in society. Nevertheless, global values are increasingly diversifying, including those regarding gender equality. Even so, universal values or aspirations that transcend Confucianism―such as learning and self-improvement, contributing to society, being considerate of one another, respecting differences, helping those in need, and adhering to rules―can be gleaned from the positive experiences voiced by Vietnamese workers. Recognizing and expanding the sharing of such values could be one pathway to realizing healthy coexistence.

### Limitations of the study

Participants were recruited via social media, which may bias the sample toward those active online and aware of workplace issues. The imbalance in residency status may limit the representativeness of each group. In addition, although responses were translated and reviewed, subtle biases in the translation process cannot be entirely ruled out. The use of free-text responses further introduces the possibility of self-selection bias, with participants holding stronger―often negative―experiences being more inclined to respond in detail. These factors may affect the generalizability of the findings.

## Article Information

### Acknowledgments

We express our profound gratitude to all participants of the survey. We also express our gratitude to Akane Futami, who passed away on March 17, 2023, for her contributions to this study.

### Author Contributions

Akihito Uezato: project conceptualization, project management, analysis, manuscript contributions; Nonoka Yoshizawa, Yui Fukuda, and Atsuko Taguchi: data collection, analysis, manuscript contributions; Pham Nguyen Quy and Tadashi Yamashita: project conceptualization, participants recruitment, manuscript contributions; Soi Jeong, Takato Komatsu, Ken Suzutani, and Akane Hayakawa manuscript contributions.

### Conflicts of Interest

None

### IRB Approval Code and Name of the Institution

The ethical review board of International University of Health and Welfare approved the study protocol (No. 19-Io-183).

## Supplement

Supplementary Material
