# Supplementary material for: Voices of Vietnamese Workers in Japan: Content Analysis Using Free-Text Responses
Source: JMA J. 2025 Nov 21;9(1):124–33. doi: 10.31662/jmaj.2025-0399 (PMC12889150; doi:10.31662/jmaj.2025-0399)
Supplement: Supplementary Material [file 2433-3298-9-1-0124-s001.pdf]

Supplementary Tables

Supplementary Table 1. Representative Participant Quotes for Each Positive Subcategory

**1. Satisfaction with work**
(1) Gained valuable learning experiences<br>"*I want to contribute to Japan because my time here has allowed me to grow. The Japanese people have taught me so much, and I've had many wonderful experiences in my life* (Specified Skilled Worker)."
(2) Work is comfortable relative to income<br>*"There's no violence or verbal abuse, and the work feels comfortable for the income I get* (ESI)."
(3) Individual employees fulfill their responsibilities<br>*"I am grateful that my colleagues take responsibility and fulfill their work* (ESI)."
(4) Work contributes to society<br>*"I am glad that I can contribute to society through my work* (Permanent Resident)."
**2. Adequate salary and rewards**
(5) Receiving appropriate compensation<br>*"The pay at my current company is fair* (ESI)."
(6) Being able to send remittances to family<br>*"Life in Japan feels very boring and repetitive, but I'm able to gain many skills and experiences. I'm also learning to be independent and can send money home to my family* (TIT)."
**3. A Conducive working environment**
(7) Work is supported by equipment<br>*"It's a good environment with high-tech equipment* (TIT)."
(8) The environment is safe and clean<br>*"The workplace is safe and clean, with various equipment and machines helping with the work. Life here is very comfortable* (Nursing care)."
**4. Good interpersonal relationships and communication in the workplace**
(9) Colleagues willingly offer support<br>*"The salary is a bit low, but there are many kind people who are happy to help when I'm in trouble, and there's no discrimination against foreigners* (Specified Skilled Worker)."<br>*"Even when the work is tough, I'm lucky to have colleagues who show mutual care and consideration. That's why, despite the challenges, I want to work hard to be of help to others* (Nursing Care)."<br>*"My colleagues are very nice, friendly, and enthusiastic. They always help me when I'm in trouble. I want to improve my Japanese skills so I can communicate better with the people at work* (Student)."
(10) Not subjected to unfair treatment<br>*"The pay is a little low, but many people are kind and willing to help when I'm in trouble, and there's no discrimination. My colleagues are interested in Vietnam and also teach me about Japanese culture* (Specified Skilled Worker)."

(11) Colleagues show mutual care and consideration<br>*"I’ve come to realize that relationships with people are the most important. Even when the job is hard, I’m lucky to have colleagues who look out for each other* (Nursing Care).*"*
(12) Having someone to consult with<br>*"I have colleagues at work I can talk to about job-related issues. Most Japanese people are polite and friendly, and they’re happy to help when asked* (Nursing Care).*"*
(13) Overcoming cultural differences.<br>*"The workplace is multicultural, and at first there were some misunderstandings. After working together for a while, we began sharing knowledge and how to do things* (ESI).*"*
**5. Positive impacts on personal life**
(14) Enjoying recreational activities with colleagues to relieve stress<br>*"In my free time, I play sports or spend time with colleagues to unwind and relieve stress* (ESI).*"*
**6. Miscellaneous**
(15) The employer complies with labor laws<br>*"I feel lucky to work at a company that properly follows labor laws* (ESI).*"*

Supplementary Table 2. Representative Participant Quotes for Each Negative Subcategory

**1. Challenges and dissatisfaction with work**
(1) Being assigned work unfairly<br>*"Japanese people hate foreigners. … I was forced to do tough work for the first time. Vietnamese workers are made to do jobs that Japanese people don’t want to do. … Since coming to Japan, I’ve grown to dislike Japanese people* (TIT).*"*<br>*"I always have to do more work than Japanese people, and it’s very stressful* (TIT).*"*
(2) Work is overly demanding<br>*"Despite paying a significant amount to come to Japan, I had to work at a company with low pay and hard labor* (TIT).*"*<br>*"The work is extremely difficult and exhausting. It makes me feel miserable and sad* (TIT).*"*
(3) Frequent exposure to work pressure<br>*"I’m under so much pressure that I feel very tired. I’ve thought about returning home many times, but I’m bound by my contract and can’t leave. If I had the chance to choose a country to work in again, I would never choose Japan* (Student).*"*<br>*"Japan’s work culture is probably world-famous, but working with Japanese people makes me feel pressured, especially regarding meeting deadlines. I feel tense and pay extra attention to avoid making mistakes* (ESI).*"*
(4) Job duties differ from expectations<br>*"I was told I’d work in industrial packaging, but when I came to Japan, I was made to carry and transport wood. When I brought this up with the labor union, they said they couldn’t help me and called me a nuisance. When I spoke*

to the Japanese staff, I was sent back to Vietnam. I feel powerless in Japan (TIT)."   *"The job I was told about during the interview was completely different from reality* (ESI)."
(5) Mechanical and inflexible work methods   *"Work is very procedural and mechanical. When something unexpected happens, it's hard to handle it flexibly.* (Nursing Care)"
(6) Limited work assignment   *"I joined a company that didn't have much work. I had to keep taking time off, so my pay is really low* (TIT)."
(7) Unsafe working conditions   *"The work is dangerous. Of the two Japanese people working with me, one tore a knee ligament, and the other fractured a rib* (TIT)."   *"We weren't taught how to ensure safety at work when we joined. I've seen numerous work-related accidents happen at these companies* (ESI)."
**2. Insufficient salary and rewards**
(8) Low wages   *"I spent over 1 million yen to come to Japan, but the salary is low. I feel exploited and disappointed* (TIT)."   *"I hope foreign workers' income will increase. Working in Japan feels like being just another machine* (TIT)."
(9) Unpaid or insufficient wages   *"I sometimes work overtime, but there is no overtime pay* (TIT)."   *"On rainy days, I don't get paid* (TIT)."
(10) Wage disparities in the workplace   *"Even though I often work harder than Japanese colleagues, my pay is lower. Sometimes it makes me not want to work* (TIT)."
(11) Inability to save due to Japan's economic downturn   *"Currently, the yen has dropped significantly, and my monthly expenses are high, leaving me with very little savings. Under such circumstances, I won't choose to stay in Japan long-term* (ESI)."
(12) Lack of access to welfare benefits   *"The job is stable, but many welfare benefits have been cut, which has dampened workers' motivation* (Specified Skilled Worker)."
(13) Exclusion from shifts with better pay   *"Our shifts were very uneven compared to Japanese workers. On bonus days, the competition for shifts was tough, and we had to ask many times before finally getting 1 or 2 shifts* (TIT)."
(14) No salary increases   *"I work hard on the tasks assigned by the company, but I felt discouraged because my pay doesn't go up* (TIT)."
**3. Inadequate rest**
(15) Forced to work overtime

"*Foreign workers often face pressure both at work and in daily life. We're discriminated against, and if we want to leave on time, we're questioned or forced to work overtime* (Specified Skilled Worker)."
(16) Poor management of break times<br>"*Foreigners often face pressure at work and in daily life, and experience discrimination. When we say we want to leave on time, we are asked why or pressured to stay for overtime. Japanese workers can take breaks even when working late, but foreigners cannot. I've raised my concerns with my bosses many times, yet nothing has changed* (Specified Skilled Worker)."
(17) Unable to rest despite illness or fatigue<br>"*I'm not allowed to take time off when I'm sick or tired, and they don't help me with paperwork. The Japanese coworkers I work with know that foreigners are treated unfairly, but they didn't speak up* (ESI)."
(18) Long working hours<br>"*Even if Vietnamese workers do better than Japanese workers, they still get paid less. The working hours are very long* (ESI)."
(19) Insufficient personal time due to busy schedules<br>"*Every day, I just go to work and keep busy like a robot, and life feels dull and unmotivated. I hardly have any friends in Japan, and no neighbors at all. I don't talk to anyone, so I feel left out even in a crowd. I go to work, come home, and don't talk to anyone—feeling almost like a recluse. Luckily, I have a partner; without them, I think I would have become depressed* (Specified Skilled Worker)."
**4. Difficulties with workplace relationships and communication**
(20) Being treated unfairly due to being Vietnamese or a foreigner<br>"*When there's an issue between foreign workers and Japanese people, they always side with the Japanese. To the company I work for, we're just cheap labor—nothing more, nothing less* (Specified Skilled Worker)."<br>"*If a Vietnamese worker makes a mistake, it's announced during the morning meeting and mocked by the Japanese staff. But when a Japanese person makes a mistake, it's ignored. We may be Vietnamese, but we are human too* (TIT)."
(21) Lack of respect for one's presence or opinions<br>"*No one listens to my opinions. Supervisors' opinions are respected, but foreign workers receive no attention* (Nursing Care)."<br>"*I am suffering from bullying* (ESI)."
(22) Challenges in communication and relationship-building due to language and cultural differences<br>"*Because of cultural differences and age gaps, I often find it hard to speak up. Although I feel secure in my job, I have to be extremely careful about what I say or answer, so it's not an environment where I can work freely and easily* (ESI)."<br>"*I try hard to get along with my colleagues, but Japanese people don't express their thoughts. I have no idea what they think of me, so I've lost the desire to talk to them* (ESI)."
(23) Mismatched dynamics with supervisors

"Working in Japan is very stressful. I think it's because my boss's personality doesn't fit me and the cultures of the two countries are different. I get bullied quite often (ESI)*."*
(24) Experiencing violence<br>*"The manager often showed disdain toward us because we're not Japanese. I experienced sexual harassment and was threatened with a knife* (Specified Skilled Worker)*."*
(25) Witnessing unfair treatment of others<br>*"I haven't had any bad experiences, but the Vietnamese people around me often get bullied, and it makes me feel bad* (TIT)*."*
**5. Negative impact on private life**
(26) Insufficient private time due to being busy<br>*"I wake up, go to work, and only close my eyes to sleep. Day after day, it's just work—like being a robot. Life feels dull and unmotivating* (Specified Skilled Worker)*."*<br>*"I'm so busy that I don't have time to have fun* (Student)*."*
(27) Loss of personal freedom and privacy<br>*"Please give us the right to live and protect our privacy. Don't impose the Japanese way of living on us. We are human too. Give us freedom and equality. It shouldn't matter where we go or who we live with as long as it doesn't affect Japanese people's work. Let us live freely and securely like you do* (TIT)*."*
**6. Miscellaneous**
(28) Lack of support or appropriate assistance in situations requiring help<br>*"Japanese colleagues know that foreign workers are treated unfairly, but they don't speak up* (ESI)*."*<br>*"When workplace injuries occur, we rarely receive proper compensation. Companies often hire lawyers to craft policies that shift the blame onto us, forcing us to admit fault so they can avoid responsibility. The Japanese judicial system often protects the interests of Japanese companies. I wish there was an organization that truly cared about the health, rights, and interests of foreign workers like us* (ESI)*."*

ESI, Engineer/Specialist in Humanities/International Services; TIT, Technical Intern Trainees
